# An Introduction to the Native and Non-Native Plant-Insect Interactions and Potential Pollinators of Puerto Williams and Yendegaia, Cabo de Hornos, Chile

**DOI:** 10.17912/micropub.biology.001158

**Published:** 2024-04-11

**Authors:** Carmen A Burkett, Ri Corwin, Jonathan Lautenbach, Issabella Serrani Gallego, Sara A Joseph, Francesca Burkett, Benton J Hendrickson, Desiree A Jackson, Morghan McCool, Clarissa M Molina, Felipe Armijo Morales, Erin R Todd, Roy Mackenzie, Laura Sanchez Jardon, Michael Thompson, Ricardo Rozzi, Andrew J Gregory

**Affiliations:** 1 Department of Biological Sciences, University of North Texas, Denton, Texas, United States; 2 Department of Biological Sciences, Northern Arizona University, Flagstaff, Arizona, United States; 3 Ecosystem Science and Management, University of Wyoming, Laramie, Wyoming, United States; 4 School of Earth Systems and Sustainability, Southern Illinois University, Carbondale, Illinois, United States; 5 The Meadows Center, Texas State University, San Marcos, Texas, United States; 6 Department of Biology, University of Louisville, Louisville, Kentucky, United States; 7 Cape Horn International Center, Puerto Williams, Chile; 8 School of Forestry, Northern Arizona University, Flagstaff, Arizona, United States; 9 Cape Horn International Center, Universidad de Magallanes, Punta Arenas, Region of Magallanes, Chile; 10 Department of Philosophy and Religion, University of North Texas, Denton, Texas, United States; 11 Sub-Antarctic Biocultural Center, University of North Texas, Denton, Texas, United States

## Abstract

The Magellanic sub-Antarctic ecoregion of southern Chile represents one of the last remaining pristine areas on Earth, but there are knowledge gaps concerning the biodiversity and interactions of the regions’ flora and fauna. Non-native insect species like *Bombus terrestris* and *Vespula vulgaris* are known to have detrimental influence on native populations through competition for resources/nesting habitat, larvae predation, and foreign pathogen introduction. However, their interactions with the native and non-native plants in the region and between introduced species are unknown. This study highlights the importance of further investigations documenting the region’s biodiversity, native and non-native species interactions, and local pollinators.

**Figure 1. Observed sub-Antarctic floral visitation map and list of floral visitors:**
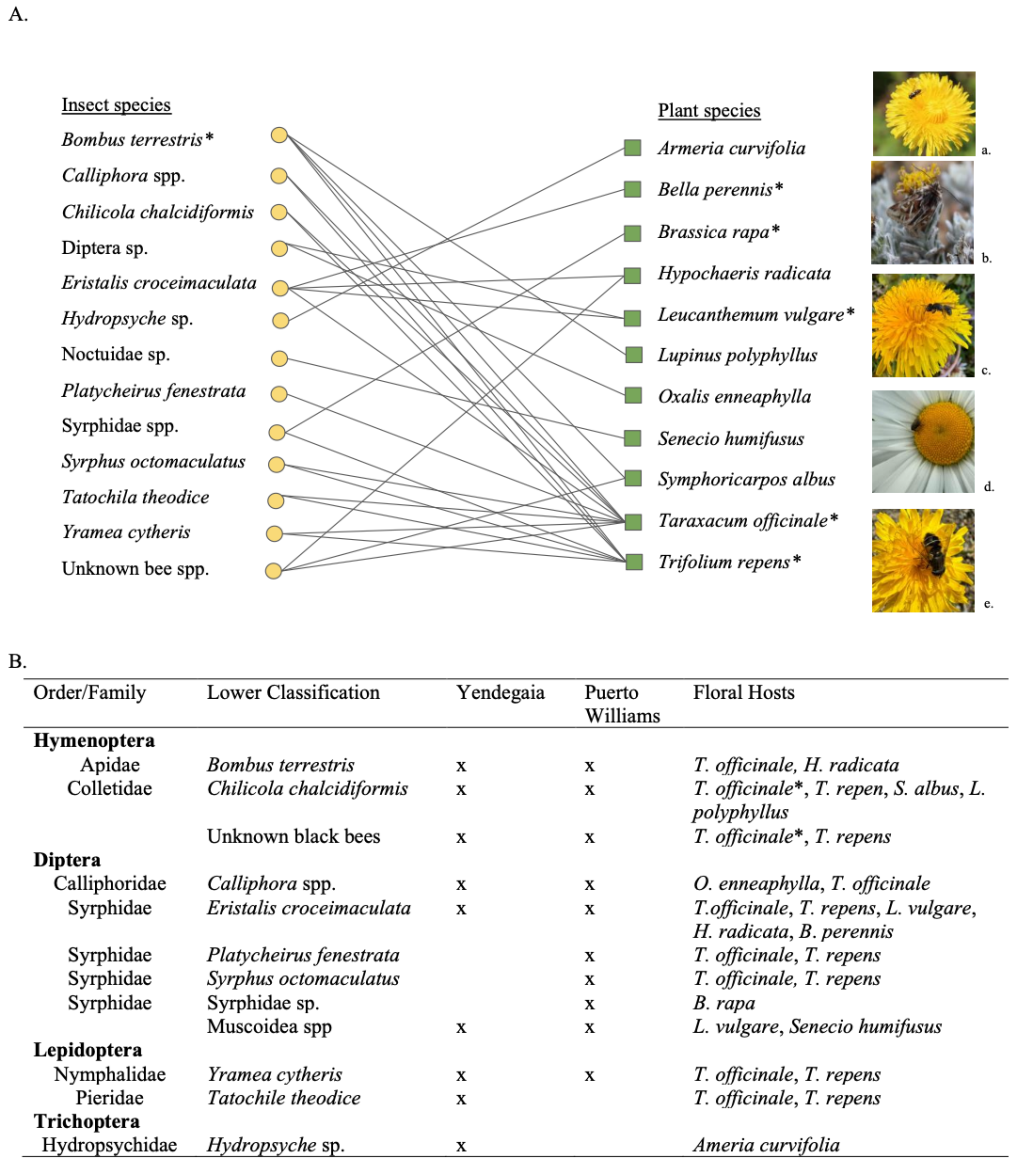
**A.** Preliminary network depicting connections between floral visitors (insects) and their floral hosts (plants) from observations taken during a two-week field intensive survey from January 1 ^st^ -16 ^th^ , 2024 (austral summer) in the Magellanic sub-Antarctic region of Chile. On the visitation map, yellow circles represent insect taxa, green squares represent plant taxa, and any lines connecting them indicate that at least one interaction between those two groups was observed. Interactions between the same insect and plant taxa may have been observed multiple times. * Denotes introduced plant and insect taxa. Photos show: a) *Syrphus octomaculatus* visiting *Taraxacum officinale* , b) Noctuidae sp. visiting *Senecio humifusus* , c) *Chilicola chalcidiformis* visiting *Taraxacum officinale* , d) Muscoidea sp. visiting *Leucanthemum vulgare,* e) *Eristalis croceimaculata* visiting *Taraxacum officinale* . Photo credit: Jonathan Lautenbach (c, d) and Carmen Burkett (a, b, e). **B.** Floral visiting insects observed, the locations in which they were observed, and their recorded floral hosts.

## Description

The Magallanes region represents a unique ecosystem and one of the last pristine habitats in the world. It contains a wealth of native biodiversity, but also includes areas where many non-native plants and animals have been introduced (Armesto et al. 2009, Rendoll-Cárcamo et al. 2017, Rendoll-Cárcamo et al. 2020, Rosenfeld et al. 2023). In at least some cases, these introduced invaders are harmful and rapidly spreading to the point that native species are disappearing (Rendoll-Cárcamo et al. 2017, Rendoll-Cárcamo et al. 2020). However, there does not currently exist a full survey of the current diversity of the region, particularly in regard to insects, or any analysis of the full effects of these non-native introductions. Also, due to the remoteness of the area and the relative recent presence of some invasive species such as the Buff-Tailed Bumblebee ( *Bombus terrestris* ), there is a lack of information regarding the interactions between native species and non-native species.

In addition, given the rapid expansion of human settlements in the area, many natural areas are being converted to novel ecosystems, which could result in a rise of dark diversity (Larson et al. 2014, Nichter & Gregory 2018) . Dark diversity represents biodiversity that is supported by what are often marginal or heavily impacted areas outside of monitored reserves. This could be, for example, in the form of gardens, prairie strips around agricultural fields, roadside habitat, or urban green spaces. A more insidious form of dark diversity may also be occurring when these marginal areas promote the persistence of non-native or potentially invasive species. For example, clover ( *Trifolium* sp.) and dandelions ( *Taraxacum* sp.) growing along roadsides and in yards and the Lupine ( *Lupinus* sp.) growing in private gardens provide floral resources for the invasive *Bombus terrestris* both in Puerto Williams and Punta Arenas ( *personal observation* ). This observational study represents, to our knowledge, the first systematic survey into the interactions among plants and native and non-native insects and potential pollinators of the region.

*Bombus terrestris* was first introduced to the Magallanes region in 2011 for agricultural use as a pollinator of red currant ( *Ribes rubrum* ). It has spread quickly since its original introduction and was first recorded on Navarino island in 2016. The Common Wasp ( *Vespula vulgaris* ) has also been recently introduced into the region and, like *Bombus* *terrestris* , is rapidly spreading. Both species may have detrimental influence on the native insect populations through competition for resources and nesting habitat, predation of larvae, and the introduction of foreign pathogens (Rendoll Cárcamo et al. 2017, Rendoll Cárcamo et al. 2022). Exotic pollinators such as *Bombus terrestris* may also contribute to the integration of non-native plant species into existing local plant communities (Parra-Tabla & Arceo-Gómez 2021).

During a 2017 survey of regional insects, Rendoll-Cáracamo et al. (2017) found no records of *Bombus terrestris* in Ushuaia on Tierra del Fuego. At the time of our surveys, *Bombus terrestris* was not only present, but also quite abundant at Yendegaia on Tierra del Fuego. Meanwhile, the region’s native Patagonian Bumble Bee, which is now endangered (Henríquez-Piskulich 2020), *Bombus dahlbohmii* , was not observed at either Puerto Williams on Isla Navarino or Yendegaia during our surveys.

Non-native plants may also affect native bee assemblages and existing plant-insect interaction networks (Henríquez-Piskulich et al. 2018, Richard et al. 2018, Parra-Tabla & Arceo-Gómez 2021). White Clover ( *Trifolium repens* ) and Dandelion ( *Taraxacum officinale* ) are both species native to Europe, however, they can be found quite abundantly in both Puerto Williams on Isla Navarino and in Yendegaia on Tierra del Fuego. Nearly all the floral visiting insects we recorded were observed visiting either one or both of these plant species, and in many cases, these were the only floral hosts on which we observed the insects, whether they were native or non-native. For example, most of our observations of *Chilicola chalcidiformis* which is a bee species endemic to Chile, occurred during visits of the species to *Taraxacum* *officinale* . Other non-native flowering plant species that were observed in the area include: *Cerastium arvense* , *Papaver* sp., and *Achillea millefolium* . The full extent of these and other non-native plant species’ interactions with native bees and other floral visitors and their effects on the native plant community is currently unknown.

Our preliminary visitation network ( Fig. 1 ) represents insect species that were seen physically on a flower and the flower species that they visited. This network simply illustrates visitation and does not represent any pollination interactions as many insect species visit flowers to nectar or rest without actually performing any pollination services to the plant (Burkett et al. 2023, Barrios et al. 2016) . Insects observed not physically visiting a flower (including *Dotocryptus bellicosus* , Anthomyiidae sp., Larentiinae sp., Noctuidae spp., *Hypodynerus vespiformis* , *Ceroglossus suturalis* , *Palpibracus chilensis* , *Aegohinus vitulus* , Anthomyiidae sp., *Vespula vulgaris* , *Aegorhinus vitulus* , and *Syncirsodes* sp.) or other flowering species in the area that were not observed having floral visitors (including *Anemone multifida* , *Erigeron* sp., *Ranunculus peduncularis* , *Perezia recurvata* , *Geranium* sp., *Adenocaulon chilense* , *Caltha sagittata* , *Senecio* sp., *Primula magellanica* , *Armeria curvifolia* , *Rubus geoides* , *Acaena ovalifolia* , *Cerastium arvense* , *Leptinella scariosa, Senecio smithii* , *Olsynium biflorum* , *Papaver* sp., *Chiliotrichum diffusum* , *Gavilea* sp., *Viola reichei* , *Codonorchis lessonii* , *Achillea millefolium* , and *Sisyrinchium patagonicum* .) are also not reflected. In only two weeks of observations, we recorded possible observations of species that are either reportedly rare in the region or previously unrecorded ( *Corynura* sp., and *Platycheirus* ( *Carposcalis* ) *walkeri* ); however, further collection and taxonomic work will be needed to confirm their presence in the region. Also, in some cases, the photographic evidence collected lacked the resolution to make a species-level identification of the floral visitor. Further sampling is needed to thoroughly assess the native insect assemblages, abundance of non-native plant and insect species, the interactions between both the native and non-native plants and insects in the region, and to determine if any of these visitors are successfully acting as pollinators.

## Methods

Floral visitors and insects belonging to taxa commonly observed as floral visitors/pollinators were observed and recorded by photos and/or videos during two weeks of the austral summer in 2024 (Jan 1st-15th). All observations were made opportunistically in each area during the approximately 14 available daylight hours of that two-week period. Observations took place in the Omora Ethnobotanical Park, throughout the town of Puerto Williams both on Isla Navarino, Cabo de Hornos, Chile, and at Yendegaia National Park in Tierra del Fuego, Chile. These floral visitors and their floral hosts were then identified to the lowest taxonomic rank possible, and records were made of the number of floral species visited by each type of floral visitor ( Fig. 1 ). We did not quantify plant and insect abundance due to the brief and highly focused sample period.

## References

[R1] Armesto JJ, Smith-Ramírez C, Carmona MR, Celis-Diez JL, Díaz IA, Gaxiola A, Gutiérrez AG, Núñez-Avila MC, Pérez CA, Rozzi R. 2009. Old-Growth Temperate Rainforests of South America: Conservation, Plant–Animal Interactions, and Baseline Biogeochemical Processes. In: Wirth C, Gleixner G, Heimann M, editors. Old-Growth Forests. Vol. 207. Berlin, Heidelberg: Springer Berlin Heidelberg. (Ecological Studies). p. 367–390. [accessed 2024 Jan 13]. http://link.springer.com/10.1007/978-3-540-92706-8_16.

[R2] Barrios B, Pena SR, Salas A, Koptur S (2016). Butterflies visit more frequently, but bees are better pollinators: the importance of mouthpart dimensions in effective pollen removal and deposition.. AoB Plants.

[R3] Burkett C. 2023. Assessing the efficacy of lepidopterans as pollinators in a midwestern pollination network [MS thesis]. Carbondale, Southern Illinois University. 269 p. ProQuest Dissertations Publishing. 29994521.

[R4] Castilla JC, Armesto JJ, Martinez-Harms MJ, Tecklin D. 2024. Conservation in Chilean Patagonia: Assessing the state of knowledge, opportunities,and challenges. Springer Cham. 504 p. [accessed 8 Feb 2024]. https://link.springer.com/book/10.1007/978-3-031-39408-9

[R5] Henríquez-Piskulich P, Vera A, Sandoval G, Villagra C (2018). Along urbanization sprawl, exotic plants distort native bee (Hymenoptera: Apoidea) assemblages in high elevation Andes ecosystem.. PeerJ.

[R6] Henríquez-Piskulich P, Vera A, Villagra C. 2020. Native bees of high Andes of Central Chile (Hymenoptera:Apoidea): biodiversity, phenology and the description of a new species of *Xeromelissa* Cockerell (Hymenoptera:Colletidae:Xeromelissinae.PeerJ 8:e8675 http://doi.org/10.7717/peerj.8675 10.7717/peerj.8675 PMC7050550 32161691

[R7] Larson JL, Kesheimer AJ, Potter DA. 2014. Pollinator assemblages on dandelions and white clover in urban and suburban lawns. Journal of Insect Conservation. 18:863-873. Doi:10.1007/s10841-014-9694-9

[R8] Nichter AN, Gregory AJ. 2018. Distribution modeling of *Asclepias spp* . predicts potential conservation benefits of marginal ecosystem as nature reserves in Northwest Ohio. Natural Areas Journal 38(4):250-258: doi.org/10.3375/043.038.0405.

[R9] Parra-Tabla V, Arceo-Gómez G (2021). Impacts of plant invasions in native plant-pollinator networks.. New Phytol.

[R10] Perez V. 2013. Introducción de *Bombus* ( *Bombus* ) *terrestris* (Linnaeus, 1758) (Hymenoptera: Apidae) en la región de Magallenes: potencial riesgo para las abejas nativas. Anales Instituto Patagonia (Chile). 41(1):147-152

[R11] Rendoll-Cárcamo J, Gañan M, Mackenzie R, Troncoso S, Troncoso J, Contador T, Rozzi R, Convey P. 2020. Macroinvertebrados dulceacuícolas del Parque Nacional Yendegaia, Chile: resolviendo brechas de conocimiento sobre la biodiversidad de la Reserva de la Biosfera Cabo de Hornos. Anales Instituto Patagonia (Chile).48(3):23-27

[R12] Rendoll-Cárcamo J, Convey P, Gañán M, Maldonado-Márquez A, Menares Zúñiga L, Contador T. 2022. Ecological features of exotic Vespula wasps (Hymenoptera: Vespidae) invading the southernmost UNESCO Biosphere Reserve. Biol Invasions. 24(7):2103–2112. doi:10.1007/s10530-022-02765-y.

[R13] Richard M, Tallamy DW, Mitchell AB. 2019. Introduced plants reduce species interactions. Biological Invasions. 21:983-992. https://doi.org/10.1007/s10530-018-1876-z

[R14] Rosenfeld S, Maturana CS, Gañan M, Rendoll Cárcamo J, Díaz A, Contador T, Aldea C, Gonzalez-Wevar C, Orlando J, Poulin E (2023). Revealing the hidden biodiversity of Antarctic and the Magellanic Sub-Antarctic Ecoregion: A comprehensive study of aquatic invertebrates from the BASE Project.. Biodivers Data J.

[R15] Rozzi R, Anderson, CB, Pizarro JC, Massardo F, Medina Y, Mansilla, AO, Kennedy JH, Ojeda J, Contador T, Morales V, Moses K, Poole A, Armesto JJ, Kalin MT. 2010. Field environmental philosophy and biocultural conservation at the Omora Ethnobotanical Park: Methodological approaches to broaden the ways of integrating the social component (“S”) in Long-Term Socio-Ecological Research (LTSER) Sites. Revista Chilena De Historia Natural. 83:000-000. Material Complementario. [accessed 2024 Jan 15]. https://digital.library.unt.edu/ark:/67531/metadc97961/

